# A Rare Case of Michelin Tire Baby Syndrome in a Newborn

**DOI:** 10.7759/cureus.2222

**Published:** 2018-02-24

**Authors:** Kamleshun Ramphul, Stephanie G Mejias, Yogeshwaree Ramphul-Sicharam

**Affiliations:** 1 Department of Pediatrics, Shanghai Xin Hua Hospital Affiliated to Shanghai Jiao Tong University School of Medicine, Shanghai, People's Republic of China; 2 Department of Pediatrics, Robert Reid Cabral Children's Hospital Affiliated to the University Iberoamericana Unibe School of Medicine; 3 Sir Seewoosagur Ramgoolam National Hospital

**Keywords:** kunze-riehm syndrome, michelin tire baby syndrome

## Abstract

Kunze-Riehm syndrome also known as Michelin tire baby syndrome (MTBS) is a rare genetic condition with a characteristic generalized folding of excess skin. The diagnosis is usually made based on clinical symptoms. There are approximately only 30 cases reported in the literature and some cases were associated with non-cutaneous anomalies as well. Herein, we report a case of MTBS in a five-day-old male of Iraqi origin.

## Introduction

Kunze-Riehm syndrome also known as Michelin tire baby syndrome (MTBS) is a rare genetic condition that consists of a characteristic generalized folding of excess skin [1]. The diagnosis is usually made based on clinical symptoms. The condition can be isolated or there can be associated extracutaneous findings as well. The pathophysiology behind this condition is still not properly understood [2] and there are approximately only 30 cases reported in the literature, and in some cases, they were also associated with noncutaneous anomalies [3]. We report a case of MTBS in a five-day-old male of Iraqi origin.

## Case presentation

**Figure 1:**
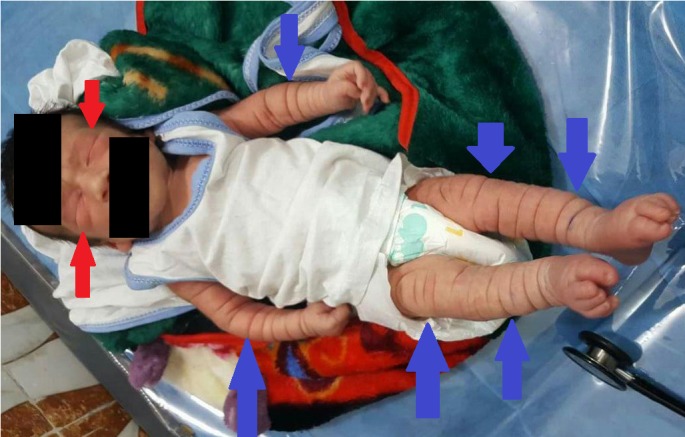
Newborn presenting with a complaint of generalized bilateral skin folding since birth, currently at day five of life in the picture Bilateral periorbital edema was observed (red arrows) as well as generalized skin folds throughout the body (blue arrows).

A five-day-old male child of Iraqi origin born from a non-consanguineous union was brought to the office for an initial complaint of generalized skin folds not regressing since birth. The pregnancy was uneventful and the mother was not compliant with prenatal care. The parents deny any family history of similar conditions. The birth weight was 2.8 kg and length 50 cm. On examination, it was observed that the child had multiple, bilaterally symmetrical, deep skin folds on the trunk as well as upper and lower extremities. Periorbital pitting edema was also detected (Figure 1).

No hypertrichosis or other cutaneous lesions were found. The patient did not have any organomegaly and the remaining systemic examination was unremarkable. Ultrasound of the abdomen and pelvis were normal as well. A diagnosis of MTBS was made. The parents refused a histopathologic examination of the skin. The child was scheduled for frequent visits to monitor the progress of the skin folds.

## Discussion

MTBS is a rare condition characterized by generalized ring-shaped skin folds. It was first described by Ross in 1969 [4] and since then about 30 cases have been reported in the literature. The origin of the name comes from the physical resemblance of these patients to the mascot of a French tire company.

Some studies have suggested a familial predisposition to the condition while others suggested evidence of an autosomal dominant profile of the condition. A case of MTBS was even observed in four generations with three male-to-male transmissions noted [5]. The pathogenesis behind this condition is still not properly understood. Deletion of the short arm of chromosome 11 has been previously reported and also a paracentric inversion of the q arm of chromosome 7 [6].

Patients of MTBS usually present with multiple generalized circumferential folds. In some cases, they are not limited to cutaneous findings; several craniofacial anomalies, cleft lip palates, and hernias have been reported [7]. Certain patients also developed psychomotor retardation as well as skeletal anomalies.

In this case, the patient presented with multiple generalized skin folds and periorbital edema. No oral anomalies were found and the systemic examination was unremarkable. There was no family history of a similar condition in the family. A 'wait and see' approach was decided and over the next few days, the periorbital edema improved but the skin folds persisted. Due to the lack of proper facilities, a karyotype analysis could not be performed and the patient was scheduled for regular check-ups.

## Conclusions

MTBS is a rare condition with multiple possible etiologies characterized by generalized skin folds. The pathogenesis is still not well understood and there can also be extracutaneous findings in some patients. The prognosis in most patients has been very positive.
